# Individual Variation in the Late Positive Complex to Semantic Anomalies

**DOI:** 10.3389/fpsyg.2012.00318

**Published:** 2012-09-06

**Authors:** Miriam Kos, Danielle van den Brink, Peter Hagoort

**Affiliations:** ^1^Radboud University Nijmegen, Donders Institute for Brain, Cognition and BehaviourNijmegen, The Netherlands; ^2^Radboud University Nijmegen, Behavioural Science InstituteNijmegen, The Netherlands; ^3^Max Planck Institute for PsycholinguisticsNijmegen, Netherlands

**Keywords:** late positive complex, inter-individual variation, event-related potentials, semantics, sentence processing, N400

## Abstract

It is well-known that, within ERP paradigms of sentence processing, semantically anomalous words elicit N400 effects. Less clear, however, is what happens after the N400. In some cases N400 effects are followed by Late Positive Complexes (LPC), whereas in other cases such effects are lacking. We investigated several factors which could affect the LPC, such as contextual constraint, inter-individual variation, and working memory. Seventy-two participants read sentences containing a semantic manipulation (Whipped cream tastes *sweet/anxious* and creamy). Neither contextual constraint nor working memory correlated with the LPC. Inter-individual variation played a substantial role in the elicitation of the LPC with about half of the participants showing a negative response and the other half showing an LPC. This individual variation correlated with a syntactic ERP as well as an alternative semantic manipulation. In conclusion, our results show that inter-individual variation plays a large role in the elicitation of the LPC and this may account for the diversity in LPC findings in language research.

## Introduction

Since its discovery (Kutas and Hillyard, 1980), the N400 has proven to be a reliable and consistent measure in the processing of meaning. Words that are semantically incongruent or have a poor semantic fit given the preceding context elicit a larger negative effect around 400 ms compared to words that fit well within the context (Kutas and Federmeier, 2011). There is, however, less consistency with respect to the Event-Related brain Potentials (ERPs) following the N400 time window. Some studies report that the N400 is followed by a late positivity or Late Positive Complex (LPC) with a broad, posterior (Münte et al., 1998; Severens and Hartsuiker, 2009; Van de Meerendonk et al., 2010; Sanford et al., 2011; Szewczyk and Schriefers, 2011) or, sometimes, frontal distribution (Federmeier et al., 2007; DeLong et al., 2011; Thornhill and Van Petten, 2012), whereas in other studies such an LPC is lacking (Osterhout and Mobley, 1995; Van den Brink et al., 2006; Van Berkum et al., 2008; Baggio et al., 2010; Stroud and Phillips, 2012; for a review see Van Petten and Luka, 2012).

Currently, it is not clear what causes this inconsistency in findings. In this study we address this issue by investigating potential triggers of the LPC. It is known that the performance of a plausibility judgment task can trigger late positivities (Kolk et al., 2003; Geyer et al., 2006; Kuperberg, 2007). However, this factor does not account for all findings, as in some experiments without an online rating task the N400 was followed by an LPC (e.g., Münte et al., 1998; Szewczyk and Schriefers, 2011), and in other ERP studies, containing an online judgment task, the LPC was lacking (e.g., Stroud and Phillips, 2012). We investigated a number of possible triggers of LPCs in two studies that did not require a response from the participant.

One potential factor that could play a role in the elicitation of the LPC is sentential constraint. Unexpected continuations in high- but not low-constraining contexts, quantified by means of cloze probability of the highest probability continuation, have been found to elicit a, predominantly frontally distributed, LPC (Federmeier et al., 2007; DeLong et al., 2011; Thornhill and Van Petten, 2012; Van Petten and Luka, 2012). A second potential trigger of the LPC is inter-individual variation, as has previously been found by Nieuwland and Van Berkum (2008). In their ERP study one group of participants showed the standard negative shift (NRef effect) to referentially ambiguous nouns (e.g., “She admired the *necklace*…” in a context with two necklaces), whereas for the other half of participants an LPC was found. Importantly, this pattern of results was related to the LPC elicited by semantically anomalous words (e.g., “She stepped into the *necklace*…”). Participants who elicited an LPC to the referentially ambiguous nouns, also showed a larger LPC to the semantically anomalous nouns as compared to the “NRef-group” (Nieuwland and Van Berkum, 2008). In addition, other experiments investigating sentence processing revealed large individual differences in late positivities as well, indicating that there are qualitative differences in the way in which subjects process sentences (Osterhout et al., 1997). Therefore, inter-individual differences in language processing could partly account for the variability of findings with respect to the LPC to semantic anomalies. Inter-individual differences in sentence processing have often been linked to differences in working memory (St George et al., 1997; Friederici et al., 1998; Gunter et al., 2003; Otten and Van Berkum, 2009; Nakano et al., 2010). Moreover, amplitude differences in the N400 effect and the LPC have also been linked to working memory variation (Van Petten et al., 1997). Therefore, inter-individual variation in the LPC may be related to working memory differences between individuals.

In the current study we investigated a number of possible underlying causes of the LPC. We did this on the basis of a substantial dataset, collected across two ERP experiments. Seventy-two participants were visually presented, amongst others, with a semantic manipulation [semantically coherent (SC): Whipped cream tastes *sweet* and creamy; semantically anomalous (SA): Whipped cream tastes *anxious* and creamy]. In addition, this dataset contained a subject-verb agreement manipulation (The spoiled child *throws/throw* the toy on the floor.), known to elicit a P600 effect (Hagoort et al., 1993). One half of the subjects was also presented with a semantic-thematic manipulation (congruent: Father eats a *sandwich/*Father eats in a *restaurant*, semantic-thematic violation: Father eats a *restaurant*/Father eats in a *sandwich*), known to elicit an N400 effect (Kos et al., 2010). This dataset gave us the opportunity to explore post hoc several factors known or hypothesized to affect the LPC. As it has been shown that contextual constraint affects the LPC (Federmeier et al., 2007; DeLong et al., 2011; Thornhill and Van Petten, 2012), we compared high versus low constraint items of the semantic manipulation. In addition, given the findings of individual differences in late positive effects in sentence processing (Osterhout et al., 1997; Nieuwland and Van Berkum, 2008), we inspected inter-individual variation within the LPC time window. Finally, we tested whether individual differences within the LPC time window were related to working memory performance (cf. Van Petten et al., 1997). Identification of factors involved in the elicitation of the LPC will contribute to a better understanding of human language processing.

## Materials and Methods

### Participants

Ninety-four highly educated native speakers of Dutch of the Donders Institute participant pool participated in one of two ERP experiments. The data of 20 of the participants were excluded from final analysis due to an excessive number of artifacts in the EEG signal, two participants were not included because of technical problems during the measurement. As a consequence, the data of 72 of the participants were included in the final analysis (36 males, mean age 21.5 years, range 18–31). All had normal or corrected-to-normal vision and were right-handed. None of the participants had any neurological or language impairment. All participants signed informed consent and received reimbursement or course credits for participation. The study was approved by the local ethics committee.

### Materials

The manipulation of interest concerned a semantic manipulation consisting of 80 Dutch sentence pairs containing a semantic violation and a correct control (Whipped cream tastes *sweet/anxious* and creamy). These sentence pairs had already been used in other experiments and are known to elicit an N400 effect (Swaab et al., 1997; Van den Brink et al., 2001; Hagoort et al., 2004). The experimental sentence pairs were identical with the exception of one word, which was the critical word for our analyses (printed in bold). Each pair consisted of a sentence that was completely semantically coherent (SC: Whipped cream tastes *sweet* and creamy) and a sentence that contained a semantic anomaly (SA: Whipped cream tastes *anxious* and creamy). The critical words were never in sentence-final position and were matched across conditions for word frequency (SC = 2.964, SA = 2.862), based on log lemma frequencies of the Dutch database CELEX (Baaijen et al., 1993), and length (SC = 5.69, SA = 5.73). The length of the sentences ranged from 5 to 19 words. The average length was 12.7 words (SD = 3.0).

Sentential constraint, i.e., the cloze probability of the highest probability continuation, was assessed by means of a behavioral experiment. Thirty-six participants, who had not participated in the EEG experiment, completed 40 sentences of these materials. Sentences were presented on a computer screen up to the critical word. Participants were asked to give one completion per item resulting in a well-formed sentence, while it was emphasized to type the first completion that came to mind. The average sentential constraint was 0.70, SD = 0.22.

In addition, both ERP experiments contained a syntactic manipulation consisting of a subject-verb agreement manipulation (The spoiled child *throws/throw* the toy on the floor), known to elicit a P600 effect (Hagoort et al., 1993). The semantic and syntactic manipulations were part of two separate ERP experiments. The two experiments had a comparable setup, which enabled us to pool the data of the semantic and syntactic manipulations, resulting in a large dataset of 72 subjects. Both experiments also contained 50 coherent items, which served as filler sentences. These coherent sentences were selected from the Dutch CLEF corpus (Van der Beek et al., 2001). In addition, we included 20 practice-items, which were similar in nature to the experimental items. For purposes which fall beyond the scope of this paper, the experiments differed with respect to the rest of the materials. One of the experiments (*n* = 36) additionally contained a set of ambiguous relative clauses and, importantly, a semantic-thematic manipulation (Father eats a *sandwich/restaurant*…/Father eats in a *restaurant/sandwich*…), known to elicit an N400 effect (Kos et al., 2010). In contrast to the semantic anomalies described above, in which the incongruent words are semantically anomalous irrespective of thematic role, the content words of the sentences of the semantic-thematic manipulation form a plausible script (e.g., father-eat-restaurant). In the thematic role violations (e.g., Father eats a *restaurant*), where in principle a syntactic alternative could be easily constructed in line with the thematic bias [*EAT* (AGENT, THEME, LOC) in which restaurant perfectly fits the LOC slot but not the THEME slot], the syntactic constraints impose a certain thematic role onto the critical word, which conflicts with its semantic-thematic role in the plausible script, resulting in an implausible scenario. Within the other experiment a set of complement clauses and a set of relative clauses (Kolk et al., 2003) were included.

Per experiment, materials were divided across lists, which were mixed pseudo randomly, with each version of each item distributed equally across the two lists, all containing an equal number of items per condition. No participant read the same sentence in more than one variant, and each variant was presented to an equal number of participants. The length of the sentences ranged from 5 to 19 words. The average length was 10.8 words (SD = 2.10; experiment1: mean = 10.3, SD = 2.26, experiment2: mean = 11.6, SD = 1.89).

### Procedure

Each participant took part in one of the two ERP experiments, consisting of one experimental session. Sentences were presented centrally using rapid visual serial presentation (both word-duration and ISI 300 ms). Participants read for comprehension in a dimly lit (Fiber optic lights DMX 512 at 60%), sound-attenuating booth. No other task demands were imposed.

After a short practice session, trials were presented in five blocks of 15 min each, separated by rest periods of approximately 5 min each. Halfway through every block there was an additional 30 s break. Viewing distance was approximately 110 cm. The first word of the sentence started with a capital letter, the rest of the words were presented in white lowercase ARIAL (23-point font size) against a dark background in the center of a CFT 60 Hz computer screen. Each word was presented for 300 ms followed by a blank screen for 300 ms. The final word of the sentence ended with a period. After the final word an asterisk appeared for 2 s, indicating to the participants that they could blink and move their eyes. There was a 1.2-s blank interval between the asterisk and the start of the next trial. Sentences were presented using Presentation software (Neurobehavioral systems, www.neuro-bs.com).

For a subset of 52 participants we assessed verbal working memory post hoc by means of a standard computerized version of the Reading Span task (mean 74.2, SD 9.8; Van den Noort et al., 2008).

### EEG recording and analysis

The EEG was recorded from 28 cap-mounted Ag/AgCl electrodes (Easycap and Acticap). Four electrodes were placed over the standard 10% system midline sites Fz, FCz, Cz, and Pz. Eleven pairs were located over the standard lateral sites FP1/FP2, F7/F8, F3/F4, FC5/FC6, FC1/FC2, T7/T8, C3/C4, CP5/CP6, CP1/CP2, P7/P8, and O1/O2. Two electrodes were placed at the outer left and right canthi to monitor horizontal eye movements. Vertical eye movements were monitored using FP1 and an electrode placed below the left eye. An additional electrode was placed on the right mastoid bone. During measurement, all electrodes were referenced to the left mastoid. For the Easycap electrode impedances of the EEG- and EOG-electrodes were kept below 5 and 10 kΩ respectively, for the Acticap electrode impedances kept below 20 kΩ. Signals were recorded with a BrainAmp DC amplifier (Brain Products, Germany), using a 125 Hz low-pass filter, a time constant of 10 s, and a 500 Hz sampling frequency. The software package Brain Vision Analyzer (Brain Products, Germany) was used to analyze the waveforms.

Offline, the EEG electrodes were re-referenced to the mean of the right and left mastoid and the EOG-electrodes were converted into bipolar horizontal and vertical EOG signals. A 30 Hz, 12 dB low-pass, Hanning filter was applied. Subsequently, the critical words were segmented using a window which started 200 ms before and ended 1500 ms after the critical word. These segments were baseline corrected to the 200 ms pre-critical-word interval. Subsequently, artifact-contaminated target trials were rejected (average 12.1%) and the remaining EEG segments were averaged per participant and per condition. Twenty participants were excluded from the analysis because more than 20% of the trials were rejected, leaving 72 participants for subsequent analysis.

Mean amplitudes of the N400 (300–600 ms) and LPC (600–1000 ms) latency windows were evaluated in repeated measures analyses of variance (ANOVA), using four quadrants (left anterior: F3, F7, FC1, FC5, C3; right anterior: F4, F8, FC2, FC6, C4, left posterior: CP1, CP5, P3, P7, O1; right posterior: CP2, CP6, P4, P8, O2), and midline sites (Fz, FCz, Cz, Pz). Whenever appropriate, Greenhouse–Geisser correction was applied. In these cases the corrected *p*-values with the original degrees of freedom will be reported.

In order to verify whether individual variation was systematic or not, we computed correlations of semantic ERP effect sizes with the subject-verb agreement manipulation (*n* = 72), known to elicit a P600 effect (Hagoort et al., 1993) and with the semantic-thematic manipulation (*n* = 36), known to elicit an N400 effect (Kos et al., 2010). These tests were conducted using Bonferroni adjusted alpha levels.

## Results

**Figure 1:**
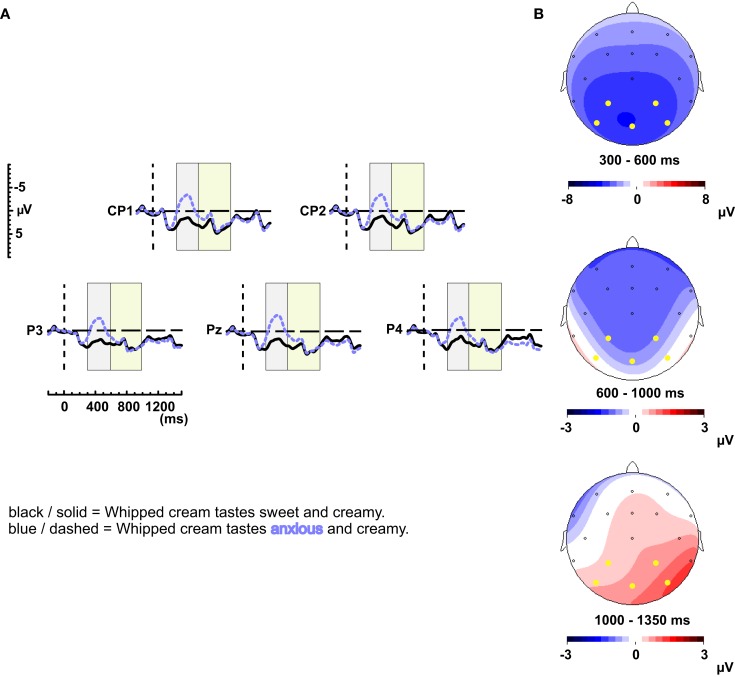
**(A)** Grand-average waveforms of the ERPs elicited by the semantic anomalies (dashed, blue line) and their correct controls (solid, black line) for electrodes CP1, CP2, P3, Pz, and P4. In this and all following figures the waveforms are time-locked to the onset of the critical word (0 ms) and negative voltage is plotted upward. Furthermore, an 8 Hz low-pass filter has been applied for illustrative purposes. The left, gray block and right, green block indicate the latency windows used for analysis for the N400 and LPC respectively. **(B)** Scalp distribution of the N400 effect elicited by the semantic manipulation between 300 and 600 ms, and of the effect between 600 and 1000 ms, and 1000 and 1350 ms after critical word onset. (In this and following figures the electrodes for which the waveforms are displayed are highlighted).

Figure 1A shows the grand-average waveforms of the semantic anomalies and the correct controls (in the absence of significant interactions between experiment and Semantic Fit, we collapsed the data over two experiments). The topographical distributions of the effects between 300 and 600, 600 and 1000, and 1000 and 1350 ms are depicted in Figure 1B. The semantic anomalies elicited a clear N400 effect. Instead of an LPC, the negativity of the N400 seems to extend into the LPC time window; only after around 1000 ms a positivity is emerging.

The repeated measures ANOVA in the 300–600 ms time window revealed a main effect of Semantic Fit [*F*(1, 71) = 137.94, MSE = 32.21, *p* < 0.001] and an interaction between Semantic Fit and Quadrant [*F*(3, 213) = 10.80, MSE = 5.12, *p* < 0.001]. Subsequent analyses show a main effect of Semantic Fit for all four quadrants [Left Anterior: *F*(1, 71) = 59.91, MSE = 12.23, *p* < 0.001; Right Anterior: *F*(1, 71) = 75.76, MSE = 10.96, *p* < 0.001; Left Posterior: *F*(1, 71) = 134.47, MSE = 11.22, *p* < 0.001; Right Posterior: *F*(1, 71) = 171.64, MSE = 8.68, *p* < 0.001]. The midline analysis revealed an effect of Semantic fit as well [*F*(1, 71) = 117.54, MSE = 10.81, *p* < 0.001]. Even though the N400 effect is widespread, the topographical distribution shows that the effect was strongest over the posterior electrodes, which is common for N400 effects (Kutas and Van Petten, 1994).

Within the LPC time window (600–1000 ms) we observed a significant negative effect of Semantic Fit across all quadrants [*F*(1, 71) = 4.96, MSE = 34.56, *p* < 0.05] and a significant interaction between Semantic Fit and Quadrant [*F*(3, 213) = 13.41, MSE = 7.59, *p* < 0.001]. Separate analyses for Semantic Fit per quadrant revealed significant negative effects for the two anterior quadrants [left anterior: *F*(1, 71) = 14.88, MSE = 11.35, *p* < 0.001; right anterior: *F*(1, 71) = 18.13, MSE = 10.10, *p* < 0.001], but no significant effects for the two posterior quadrants [left posterior: *F*(1, 71) < 1; right posterior: *F*(1, 71) < 1]. The midline analyses revealed a significant effect of Semantic Fit [*F*(1, 71) = 9.03, MSE = 13.12, *p* < 0.01].

On the basis of visual inspection we performed additional analyses between 1000 and 1350 ms after word onset to explore the late positive effect. Across all quadrants there was no effect of Semantic Fit [*F*(1, 71) = 1.33, MSE = 40.89, *p* = 0.253], but an interaction between Semantic Fit and Quadrant was found [*F*(3, 213) = 16.93, MSE = 7.60, *p* < 0.001]. Further analyses for the separate quadrants revealed a significant right posterior effect of Semantic Fit [left anterior: *F*(1, 71) = 3.33, MSE = 14.32, *p* = 0.072; right anterior: *F*(1, 71) < 1; left posterior: *F*(1, 71) = 3.16, MSE = 11.38, *p* = 0.080; right posterior: *F*(1, 71) = 13.06, MSE = 15.87, *p* < 0.01]. Semantic Fit did not reach significance at the midline electrodes [*F*(1, 71) = 2.97, MSE = 14.96, *p* = 0.089].

### Contextual constraint

**Figure 2:**
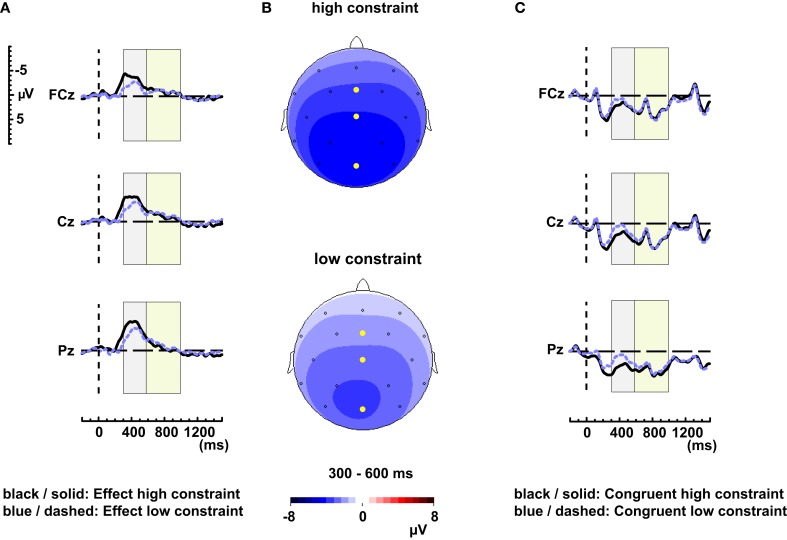
**(A)** Difference waveforms elicited by the high contextual constraint (solid, black line) and low contextual constraint (dashed, blue line) items for electrodes FCz, Cz, and Pz. **(B)** Scalp distributions of the N400 effect of the high and low constraint items. The high constraint items elicit a larger N400 effect compared to the low constraint items. **(C)** Grand-average waveforms of the congruent high (solid, black line) and low constraint (dashed, blue line) items for electrodes FCz, Cz, and Pz.

We assessed effects of contextual constraint (cloze probability of the highest probability continuation) by grouping the items based on a median split (low constraint: mean = 0.50, SD = 0.11; high constraint: mean = 0.90, SD = 0.10; incongruent critical words matched across constraint conditions for frequency [*t*(158) = 1.375, *p* = 0.17]. Figure 2A shows the difference waveforms (incongruent minus congruent) for the high and low contextual constraint items. The N400 effect (300–600 ms) of the high contextual constraint sentences is larger compared to the low contextual constraint sentences across all four quadrants (Constraint × Semantic Fit *F*(1, 71) = 9.82, MSE = 48.73, *p* < 0.01). The interaction between Constraint × Semantic Fit × Quadrant was not significant [*F*(3, 213) = < 1]. Midline analyses revealed a significant interaction between Constraint and Semantic Fit [*F*(1, 71) = 6.34, MSE = 21.81, *p* < 0.05]. Figure 2B shows the topographical distributions of the N400 effect elicited by the high constraint and low constraint items.

Within the LPC time window (600–1000) significant interactions between Contextual constraint and Semantic Fit are absent [across four quadrants: *F*(1, 71) < 1; Constraint × Semantic Fit × Quadrant *F*(3, 213) = 1.93, MSE = 6.20, *p* = 0.14; Midline: Constraint × Semantic Fit *F*(1, 71) < 1].

As previous experiments revealed differences between high and low-constraining contexts of congruent semantic words (Federmeier et al., 2007; DeLong et al., 2011; Thornhill and Van Petten, 2012), we also analyzed the ERPs of the congruent words within the high and low contextual constraint conditions (congruent critical words matched across constraint conditions for frequency [*t*(78) = 0.17, *p* = 0.86]). Figure 2C shows the waveforms to the congruent conditions for the high and low contextual constraint items. The low constraint words elicit a larger N400 component compared to the high constraint words over four quadrants and over the midline sites [four quadrants: *F*(1, 71) = 14.30, MSE = 57.25, *p* < 0.001; midline: *F*(1, 71) = 6.81, MSE = 24.22, *p* < 0.05]. The interaction between Constraint and Quadrant was also significant between 300 and 600 ms [*F*(3, 213) = 3.70, MSE = 6.88, *p* < 0.05]. Separate analyses per quadrant revealed that the differences between high and low constraint were present for all four quadrants [left anterior: *F*(1, 71) = 6.32, MSE = 19.46, *p* < 0.05; right anterior: *F*(1, 71) = 4.97, MSE = 21.20, *p* < 0.05; left posterior: *F*(1, 71) = 18.90, MSE = 15.04, *p* < 0.001; right posterior *F*(1, 71) = 21.63, MSE = 17.07, *p* < 0.001].

Analyses between 600 and 1000 ms showed no significant effects for Contextual constraint [all four quadrants: *F*(1, 71) = 2.10, MSE = 50.44, *p* = 0.151; Midline: *F*(1, 71) < 1], nor an interaction between Constraint and Quadrant [*F*(3, 213) = 1.88, MSE = 7.72, *p* = 0.150].

### Inter-individual variability LPC

**Figure 3:**
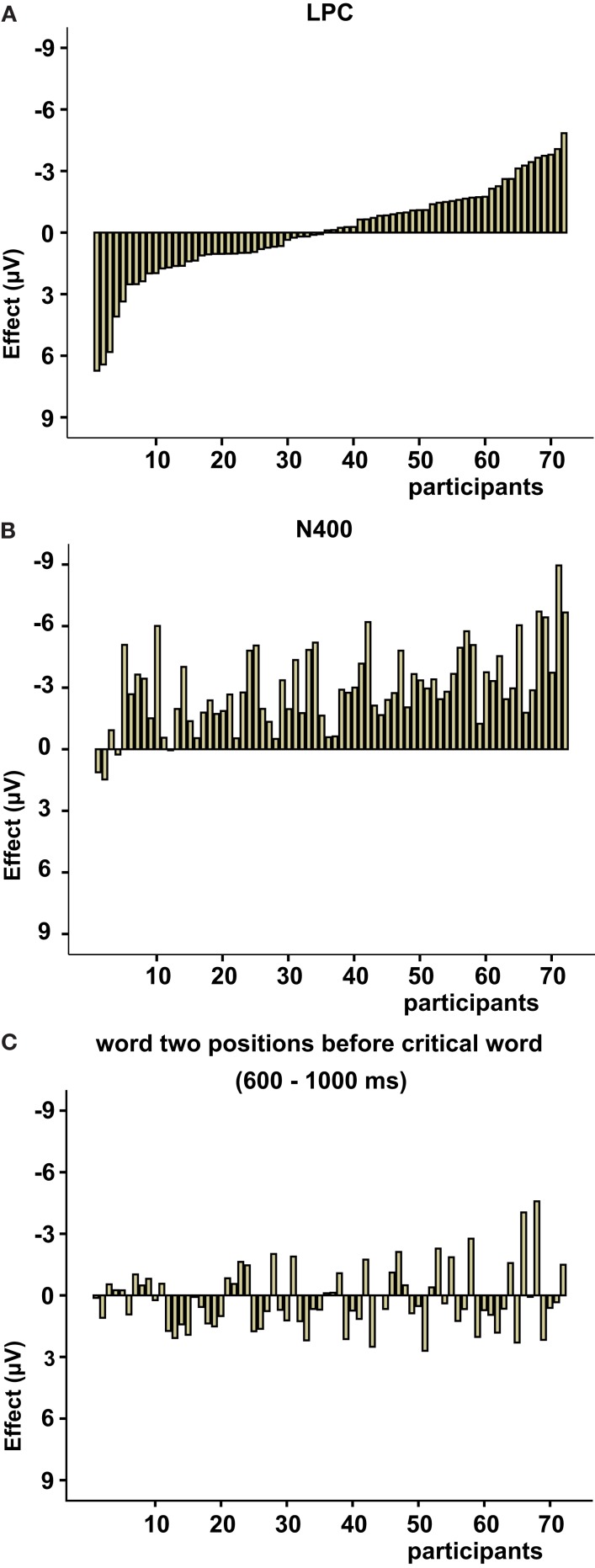
Effect sizes per participant for the semantic manipulation within **(A)** the LPC time window (600–1000 ms) and **(B)** the N400 time window (300–600 ms). **(C)** Effects per participant on the word two positions before the critical word of the semantic manipulation between 600 and 1000 ms. Participants are sorted on LPC effect size for easier comparison between the figures.

Given the posterior distribution of the positivity observed across all participants between 1000 and 1350 ms, we explored the individual effects within the standard LPC time window (600–1000 ms) across the 11 posterior electrodes (mean = −0.06, SD = 2.28). This analysis reveals the amount of individual variation within this time window with approximately half of the participants showing a negative and half a positive effect (see Figure 3A), resulting in an absence of a significant overall main effect. Inspection of the N400 time window shows that almost all participants elicit a negative effect with less overall variation (mean = −2.98, SD = 1.98; Figure 3B). To investigate whether the variation within the LPC time window could be attributed to noise, we inspected ERP effects elicited by other words within the sentence. To avoid temporal overlap or cross-over effects of semantically anomalous words, we analyzed the words that were positioned two words before the critical words used in the analyses above. Figure 3C depicts the individual ERP effects between 600 and 1000 ms after onset of the word. Also here, the effects vary across individuals (mean = 0.17, SD = 1.51), but the variation to the random words is lower compared to the variation observed for the LPC [*F*(1, 142) = 6.934, *p* < 0.01].

In order to further explore the consistency of the inter-individual variation, we performed correlation analyses with the subject-verb agreement and semantic-thematic manipulation using the individual effects between 600 and 1000 ms of the critical words and the words positioned two words before these critical words. This resulted in four tests for which Bonferroni adjusted alpha levels of 0.0125 were used.

**Figure 4:**
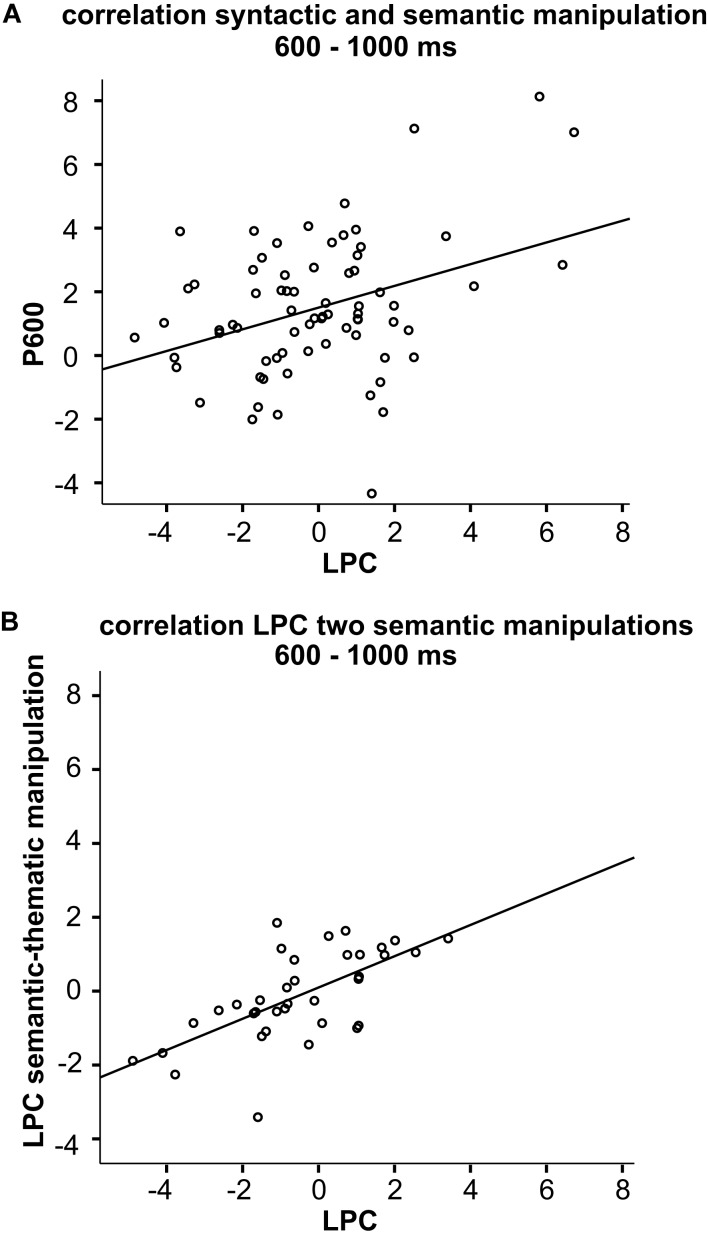
**(A)** Scatter plot of the effect of the semantic manipulation within the LPC time window (600–1000 ms) and the P600 effect elicited by subject-verb agreement violations within the same latency window, which are correlated. **(B)** Scatter plot for half of the participants who were offered two semantic manipulations. The plot shows the effects within the LPC time window. These effects are highly correlated.

Individual ERP effects elicited by the syntactic and semantic manipulation between 600 and 1000 ms were significantly correlated [*r*(70) = 0.37, *p* < 0.01; see Figure 4A]. Furthermore, individual effects within the LPC time window of the two semantic manipulations were highly correlated [*r*(34) = 0.65, *p* < 0.001; see Figure 4B]. In contrast, individual variation to the word positioned two words before the critical word did not correlate with the variation observed for the P600 [*r*(70) = −0.20, *p* = 0.10] nor with variation seen in the LPC time window of the semantic-thematic manipulation [*r*(34) = −0.10, *p* = 0.58].

**Figure 5:**
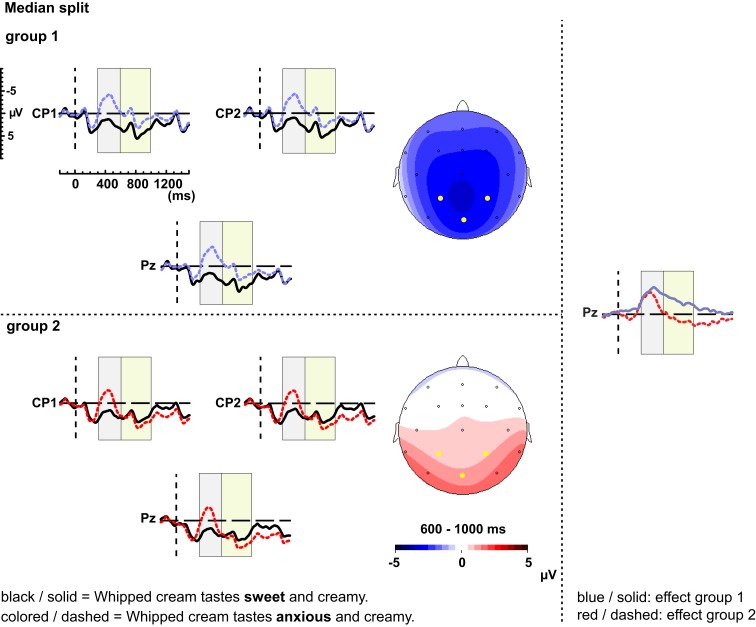
Two groups were created by means of a median split of the posterior effect between 600 and 1000 ms. The left panel of this figure shows their grand-average waveforms of the ERPs elicited by the semantic anomalies (dashed, colored line) and the correct controls (solid, black line) for electrodes CP1, CP2, and Pz. Additionally, the scalp distribution of the effect elicited by the semantic manipulation between 600 and 1000 ms is depicted. The right side displays the effect or difference waveforms of group 1 (blue, solid line) and group 2 (red, dashed line) at electrode Pz.

In order to visualize the individual variation within the LPC time window, we divided the participants based on a median split (Figure 5). One half of the participants showed an extended negativity after the N400, whereas the other half exhibited an LPC. On the right panel of Figure 5 the effects of the two groups are directly compared.

There were no relations between semantic ERP effects and reading span scores, as assessed by computing the correlation between the individual effects over posterior electrodes between 300 and 600 and 600 and 1000 ms and their reading span scores [*n* = 52; 300–600 ms: *r*(50) = −0.08, *p* = 0.56; 600–1000 ms: *r*(50) = 0.12, *p* = 0.39; *n* = 28 for the negative responders, *n* = 24 for the positive responders]. Nor can these individual differences be explained by gender [*t*(70) = −0.40, *p* = 0.69] or experimental version [*t*(70) = −0.16, *p* = 0.99]. It has been suggested that familial left-handedness is related to individual differences in language processing (Townsend et al., 2001). We retrieved information about the handedness of the parents of 70 of our participants. Fourteen out of 70 reported having at least one left-handed parent. Eleven of these 14 belong to the negative, no LPC group.

## Discussion

A substantial dataset, consisting of the ERP responses of 72 participants to a semantic manipulation (Whipped cream tastes *sweet/anxious* and creamy.), enabled us to examine potential factors involved in the elicitation of the LPC, such as contextual constraint (Federmeier et al., 2007; DeLong et al., 2011), individual variation (Osterhout et al., 1997; Nieuwland and Van Berkum, 2008), and working memory (Van Petten et al., 1997; Friederici et al., 1998). The aim of this study was to identify possible underlying causes of the LPC and to reach to a better understanding of this ERP effect. We found that, across all 72 participants, the semantic manipulation elicited a robust N400 effect. The negativity of this N400 effect extended into the LPC time window over the frontal electrodes. There was no positivity within the standard LPC time window (600–1000 ms). Instead, a posterior positivity emerges only after around 1000 ms (see Figure 1).

First, we investigated whether contextual constraint modulates ERP waveforms in the LPC time window, as previous research suggests that the LPC is related to the possible cost of processing unexpected words in strongly constraining contexts (Federmeier et al., 2007; DeLong et al., 2011; Thornhill and Van Petten, 2012; Van Petten and Luka, 2012). We found a larger N400 effect in the high constraint compared to the low constraint condition, and this is in line with previous research (Federmeier et al., 2007; Kutas and Federmeier, 2011). In contrast to earlier findings (Federmeier et al., 2007), the effects within the LPC time window did not differ between the high and low-constraining conditions. Previous experiments revealed differences between high and low-constraining contexts of congruent semantic words leading to larger N400 components and larger frontally distributed LPCs (Federmeier et al., 2007; DeLong et al., 2011; Thornhill and Van Petten, 2012; Van Petten and Luka, 2012). Therefore, we also analyzed the effect of contextual constraint in the congruent condition. Again, the findings within the N400 time window mimic previous results with the N400 component of high cloze words being smaller compared to that of the low cloze words (DeLong et al., 2011; Kutas and Federmeier, 2011). We did, however, not find any differences with respect to contextual constraint within the LPC time window. The fact that the N400-results were compatible with previous findings does suggest that our experimental procedure was successful in achieving the desired contrast between high and low contextual constraint. Therefore, the findings within the LPC time window challenge theories presupposing that the LPC is mainly associated with processes driven by semantic expectancies (Federmeier et al., 2007; DeLong et al., 2011), as they would predict a modulation of the LPC based on the strength of contextual constraint.

Another potential factor which may play a role in the elicitation of the LPC are individual characteristics of the participants, as previous research revealed individual variation in ERP effects (Osterhout et al., 1997) and even more specifically in the LPC (Nieuwland and Van Berkum, 2008). Our relatively large dataset gave us the opportunity to explore individual variation within the LPC latency range. For our 72 participants we observed substantial inter-individual variation in the LPC latency window, with about one half of the participants showing an extended negative effect and the other half exhibiting an LPC (see Figure 3A). This was in contrast to the N400 latency window in which the individual effects showed hardly any variation with respect to polarity (see Figure 3B). To get an estimate about the standard amount of noise generated by the processing of an incoming word within our paradigm, we analyzed the variation of the ERP effects of another word within the same sentence (e.g., the word two positions before the critical word). Even though we observe some variation for these words, this variation is smaller compared to the variation observed for the LPC of the critical words, indicating that the variation found for the LPC not merely reflects noise (Figure 3C). To gain further insight in whether the inter-individual variation observed for the effects within the LPC latency window was systematic, we looked at correlations with other manipulations at the individual level. We reasoned that if the variation was systematic, it should be associated with other manipulations affecting related ERP effects. Therefore, we analyzed whether the LPC and the P600 effect, elicited by a subject-verb agreement manipulation, were correlated. We found that the sizes of the individual ERP effects within the LPC time window of the semantic manipulation were indeed correlated with the sizes of the individual P600 effects. Moreover, the variation observed for the word positioned two words before the critical word of the semantic manipulation did not correlate with the syntactic manipulation.

Our dataset also contained a semantic-thematic manipulation for which an N400 effect was found (Kos et al., 2010). It is to be expected that similar processes underlie the two semantic manipulations and that the individual variation within the LPC latency window of these two manipulations is closely correlated. Statistical analyses indeed revealed that the individual ERP responses within the LPC time window of the semantic manipulation were strongly correlated with the responses elicited by the semantic-thematic manipulation. Again, the variation found for the word positioned two words before the critical word was unrelated to this semantic-thematic manipulation. These findings confirmed that the individual variation observed for the LPC is systematic and is therefore indicative of an underlying cognitive process, which differs between participants.

Previous research points to differences in working memory playing a role in the elicitation of the LPC (Van Petten et al., 1997). We did, however, not replicate the correlation between inter-individual variation within the LPC time window and working memory performance. Earlier research suggests that gender differences play a role in differences in semantic processing and the elicitation of the LPC (Daltrozzo et al., 2007), but also this factor could not account for the individual variation in the LPC. It has been suggested that familial left-handedness is related to individual differences in language processing (Townsend et al., 2001). We observed that a substantial part of our participants with one or two left-handed parents (i.e., 11 out of 14) are in the negative responders group. Handedness of family members and concomitant processing strategies may be related to the elicitation of the LPC. However, future research testing more balanced samples of right-handed participants with and without left-handed family members is necessary to draw more firm conclusions concerning this potential factor in elicitation of the LPC.

Individual variation in patterns of language-related ERPs has been reported before. Nieuwland and Van Berkum (2008) showed that some individuals elicited an LPC to referentially ambiguous nouns, whereas others did not. However, individual variation has not only been found with respect to the LPC. Also for other language-related tasks inter-individual variation has been observed, and this variation has been explained by a variety of factors. It has, for instance, been found that variation in syntactic processing is related to one’s proficiency in grammar and vocabulary (Pakulak and Neville, 2010). Ye and Zhou (2008) showed that differences in the processing of conflicting sentence representations were related to individual differences in cognitive control (quantified by performance on a Stroop task). Another sentence processing study using ERPs revealed that individual differences in verbal social information processing could be explained by individuals’ cognitive styles, related to one’s ability to empathize (Van den Brink et al., 2012). Additionally, studies investigating heritability or genetic effects reveal heritable variation in language processing across healthy individuals (Ramus and Fisher, 2009; Snijders, 2010). In sum, various individual factors may account for the inter-individual variation found for the LPC. Future ERP studies investigating semantic processing could take this variation into account when attempting to elucidate the nature of the individual differences by balancing the number of right-handed participants with and without left-handed family members, as well as obtaining additional individual behavioral measures such as measures of cognitive style or vocabulary.

With the lack of identification of an individual factor responsible for the elicitation of the LPC, we can only give tentative suggestions with respect to the functional process underlying the LPC. Partly based on findings in a recent fMRI study (Burholt Kristensen et al., 2012), we have come to realize that across a sentence depth of processing may vary. This is related to what has been referred to as “good-enough” processing (Ferreira, 2003). Violations of either type (semantic, grammatical) next to their language-related signatures (e.g., N400, P600) may also be triggers that recruit general attentional resources for more in-depth processing. Analogous to markers of Information Structure, violations could serve as triggering signals for deeper processing. The LPC is in this case a marker of (recruiting attentional resources for) deeper or extended processing, which should be distinguished from the P600. Here one can easily expect individual differences in the degree to which the violations trigger additional processing.

In conclusion, our results revealed substantial and systematic inter-individual variation within the LPC time window across a relatively large sample of subjects. In ERP studies it is common practice to average over participants and to assume that the averaged ERP waveform is indicative of similar neuronal processes in all participants. However, some ERP effects seem to be subject to inter-individual variability and in these cases the grand-average can obscure the differential underlying processes of participants. Importantly, variation between individuals may partly explain the inconsistency in results with respect to the LPC across experiments. Therefore, inter-individual variation is a factor to take into account in studies investigating language processing.

## Conflict of Interest Statement

The authors declare that the research was conducted in the absence of any commercial or financial relationships that could be construed as a potential conflict of interest.
